# Efficacy of low-frequency low-intensity electrotherapy in the treatment of breast cancer-related lymphoedema: a cross-over randomized trial

**DOI:** 10.1177/0269215511427414

**Published:** 2012-07

**Authors:** Roser Belmonte, Marta Tejero, Montse Ferrer, Josep M Muniesa, Esther Duarte, Oriol Cunillera, Ferran Escalada

**Affiliations:** 1 Medicina Física i Rehabilitació, Hospital Mar-Esperança, Barcelona, Spain; 2 Departament Medicina, Universitat Autònoma de Barcelona, Barcelona, Spain; 3 IMIM Institut de Recerca Hospital del Mar, Barcelona, Spain; 4 CIBER en Epidemiologia y Salud Pública, CIBERESP España; 5 Departament de Pediatria, Obstetricia i Ginecologia, i de Medicina Preventiva, Universitat Autònoma de Barcelona, Bellaterra, Barcelona, Spain

**Keywords:** Breast cancer, electric stimulation therapy, lymphoedema, physical therapy, quality of life

## Abstract

**Objective**: To compare the efficacy of low-frequency low-intensity electrotherapy and manual lymphatic drainage in the treatment of chronic upper limb breast cancer-related lymphoedema.

**Design**: Cross-over single-blind random clinical trial.

**Setting**: Rehabilitation service.

**Participants**: Thirty-six women with chronic upper limb breast cancer-related lymphoedema.

**Methods**: Patients were randomized to undergo 10 sessions of manual lymphatic drainage followed by 10 sessions of low-frequency low-intensity electrotherapy or to undergo first low-frequency low-intensity electrotherapy followed by manual lymphatic drainage. There was a month of washout time between treatments. Each patient was examined just before and after each treatment. Researchers and outcome assessors were blinded for assigned treatment.

**Measures**: Outcomes were lymphoedema volume, pain, heaviness and tightness, and health-related quality of life measured with the Functional Assessment of Cancer Therapy Questionnaire for Breast Cancer version 4 (FACT-B+4). Carry-over, period and treatment effects were analysed. Treatment effect was assessed using paired *t*-test.

**Results**: Thirty patients finalized treatment. Comparing the changes in low-frequency low-intensity electrotherapy with manual lymphatic drainage changes, there were no significant differences. Low-frequency low-intensity electrotherapy did not reduce lymphoedema volume (mean of change = 19.77 mL, *P* = 0.36), but significant reductions were observed in pain, heaviness and tightness (mean of change = 13.1, 16.2 and 6.4 mm, respectively), and FACT-B+4 summaries improved significantly (Trial Outcome Index mean of change = 5.4, *P* = 0.015). Manual lymphatic drainage showed no significant changes in any of the outcomes

**Conclusion**: Although there are no significant differences between treatment changes, the observed trend towards a better health-related quality of life is remarkable in low-frequency low-intensity electrotherapy.

## Introduction

Upper limb lymphoedema is an important complication after surgical treatment of breast cancer. Lymphoedema is a chronic condition that tends to progress and cause physical, functional, psychological and social morbidity. Although it cannot be cured, it can be successfully managed by complex decongestive therapy.^1^–^3^

Manual lymphatic drainage is one of the components of the complex decongestive therapy, directing lymphatic flow out of congested areas and into functional lymph node basins. It is used in the initial reductive phase, and could be used in the maintenance phase of lymphoedema treatment.^1^–^6^ Other therapies such as low-level laser or electrotherapy have been suggested to be useful modalities in the treatment of breast cancer-related lymphoedema,^7^,^8^ but there are very few studies about their effectiveness. Recently, Jahr et al.^8^ used intermittent electrostatic fields of low intensity and extremely low frequency to stimulate lymphatic flow by a deep resonance vibration. This study reported pain alleviation and swelling reduction in patients with breast lymphoedema related to breast cancer.

Ricci^9^ evaluated the effects of an electro-medical instrument which uses low-frequency and low-intensity electrotherapy to treat lymphoedema, by the activation of the biological structures contained in the lymph through the physical process of bioresonance. He applied this therapy to 50 patients, and used lymphoscintigraphy to verify the effect. The study concluded that the treatment stimulates lymph flow, activates apical limb lymph nodes and reduces dermal back flow. They reported that the low-frequency low-intensity electrotherapy system was useful in diminishing volume and the ‘feeling of gravity and hardening’. The study by Ricci provides the first published data on the effect of electric fields in upper and lower limbs lymphoedema, but there are no studies testing the efficacy of low-frequency low-intensity electrotherapy on improving signs, symptoms and health-related quality of life in the treatment of peripheral lymphoedema.

The hypothesis of how low-frequency low-intensity electrotherapy works is similar to that of manual lymphatic drainage. Both return lymph to lymphatic circulation, though low-frequency low-intensity electrotherapy could add an effect of molecular protein activation. Our objective was to compare the efficacies of low-frequency low-intensity electrotherapy and manual lymphatic drainage treatments in reducing volume, pain, heaviness, tightness and improving health-related quality of life in patients with chronic breast cancer-related lymphoedema of the upper limb.

## Methods

The study had a randomized, blind, cross-over design with two treatment phases separated by a one-month washout period. Eligible patients were randomly assigned to one of two groups: group A underwent low-frequency low-intensity electrotherapy followed by a manual lymphatic drainage period; and group B underwent first the manual lymphatic drainage followed by a low-frequency low-intensity electrotherapy period.

A computer-generated randomization list was obtained from the research service. An independent supervisor allocated eligible patients to groups A or B using this list. Patients and therapists could not be blinded because of the obvious differences between both treatments. Physicians who examined patients and data analysts were blinded for the treatment received. Patient evaluations were performed in the medical office (never in the treatment room) by two physicians who were also study investigators (RB and MT). In order to ensure that blinding was successful, patients were advised not to mention their treatment during physician evaluation, except for adverse effects.

Women with chronic breast cancer-related lymphoedema were recruited by the physician in an outpatient hospital rehabilitation setting between March 2008 and July 2009, obtaining their informed consent before randomization. The study was performed in accordance with the ethical standards laid down in the Helsinki Declaration of 1975, revised in 1983, and had previously been approved by the institutional review board.

Inclusion criteria were having breast cancer-related lymphoedema, to have finished the intensive phase of complex decongestive therapy for lymphoedema, started the maintenance phase at least a year ago, and observed a therapeutic clearance period without manual lymphatic drainage treatment for at least six months. Exclusion criteria were: (1) the presence of a pacemaker, heart disease, pregnancy, metallic devices in the limb to be treated, infectious disease, epilepsy, cartilage growth, thrombophlebitis, arterial hypertension or metastases, which are the treatment contraindications; and (2) the presence of mental, sensorial or language problems, which could make cooperation difficult.

A total of 10 sessions of manual lymphatic drainage and 10 sessions of low-frequency low-intensity electrotherapy were given to each patient, once per day from Monday to Friday. Both treatments were applied by a physiotherapist expert in lymphoedema treatment. There was a month washout period between the two treatments. Patients were required to immediately report any complication or adverse effect to the attending service. During both treatment periods, the maintenance phase of complex decongestive therapy was continued: compression therapy was continued using adapted garments (sleeves and gloves) and patients were reminded to continue exercises and skin care.

Low-frequency low-intensity electrotherapy was applied using the Flowave2Home (Talamonti Group S.r.1., Acquaviva Picena, Italy. www.flowave.it) electro-medical instrument, which works with the same system that Ricci^9^ used. It is described by the manufacturer as a massage system developed for physiological activation of the molecules composing the lymph, through microcurrent and bioresonance induced by weak, varying, low-frequency electric fields. The energetic activation of such molecules would cause their migration along the physiological channels following the principle of the path of least resistance. The treatment is effected through a wave of carrier frequency ranging from 0.31 to 6.16 Hz and a modulation between 400 and 2120 Hz; the low offset voltage is always between +12 and −12 V.

The treatment technique includes the use of a pair of maniples and eight pairs of electrodes which are applied to the patient’s skin in areas corresponding to lymphonodal stations. The application is made using a slow circular motion, without pressing, following lymphatic routes. Patients could feel a slight tingling or a slight sensation of heat during treatment, but it is absolutely painless. An upper limb lymphoedema treatment application takes about 50 minutes.

For data collection, a physician examined every patient just before and just after every 10 treatment sessions. The average pain, heaviness and tightness during the last week were measured by 100 mm visual analogue scales. The volume of both upper limbs was calculated by the method described by Taylor et al.,^10^ which showed good reliability. Briefly, this consists of determining anatomic landmarks of the upper limb, and then measuring six circumferences. The volumes are then obtained by the truncated cone formula. The lymphoedema volume was obtained by the difference between affected and unaffected upper limb volumes. Lymphoedema was classified according to severity as mild (lymphoedema volume <20%), moderate (20–40%) and severe (>40%).^2^ Physicians asked about any adverse effect of the treatment.

Health-related quality of life was measured by the Functional Assessment of Cancer Therapy Questionnaire for Breast Cancer version 4 (FACT-B+4), composed of four generic subscales (physical well-being, social well-being, emotional well-being, functional well-being), and two specific subscales (breast cancer subscale and arm subscale).^11^–^14^ FACT-General (score range 0–108) is the summary measure of the four generic subscales, and FACT-Breast (score range 0–144) is the same plus the breast cancer subscale. The trial outcome index (score range 0–92) is a summary measure of the physical and functional well-being generic scales and breast cancer subscale. The arm subscale is not used in the calculation of any summary measure. Higher FACT scores indicate better health-related quality of life. Eton et al.^15^ estimated the following minimally important differences: FACT-General: 5–6 points, breast cancer subscale: 2–3 points, FACT-Breast: 7–8 points, and trial outcome index: 5–6 points.

The descriptive variables collected were age, weight, height, body mass index (kg/m^2^), surgical treatment, axillary resection, chemotherapy, radiation therapy, duration and severity of lymphoedema, affected side, history of infections or other complications, and use of compression garments.

The sample size calculation, with an overall power of 80% to detect differences in the primary end-point (lymphoedema volume), was based on: a low-frequency low-intensity electrotherapy benefit estimated at 120 mL reduction following the standard error of measurement calculated by Sander et al.,^16^ a manual lymphatic drainage benefit estimated at 70 mL reduction as obtained in the Williams et al.^17^ trial, and the standard deviation of the difference between these reductions estimated at 100. A total sample of 34 patients was required to detect this 50 mL difference between treatments effects using a paired *T*-test (two-tailed, type I error rate of 5%).

The mean and standard deviation for continuous variables, and frequency distribution (percentage) for categorical data was used for descriptive analysis.

The primary outcome was the change in the lymphoedema volume, and secondary outcomes were pain, heaviness, tightness and health-related quality of life. Analyses of the primary and secondary efficacy outcomes were performed by testing three effects: First, the carry-over effect considers whether the impact of the first treatment is still present when the patient enters the second treatment period. The mean (and 95% confidence interval) of the difference between the evaluation at baseline and the evaluation at the end of the washout period was estimated to assess this carry-over effect. Second, the period effect considers whether the impact of low-frequency low-intensity electrotherapy treatment was different when the order of administration changed. The period effect was evaluated by comparing the mean change difference between treatments in group A and group B using an unpaired *t*-test. Third, the treatment effect considers the benefit of manual lymphatic drainage and low-frequency low-intensity electrotherapy, which were estimated as the mean change pre-post treatment in the total sample. Treatment effect of low-frequency low-intensity electrotherapy and manual lymphatic drainage were assessed and compared using a paired *t*-test. All reported *P*-values were two-tailed. A type I error rate of 5% was used. Data was analysed using the SPSS version 12 statistical package (SPSS Inc., Chicago, IL, USA).

## Results

**Figure 1.**
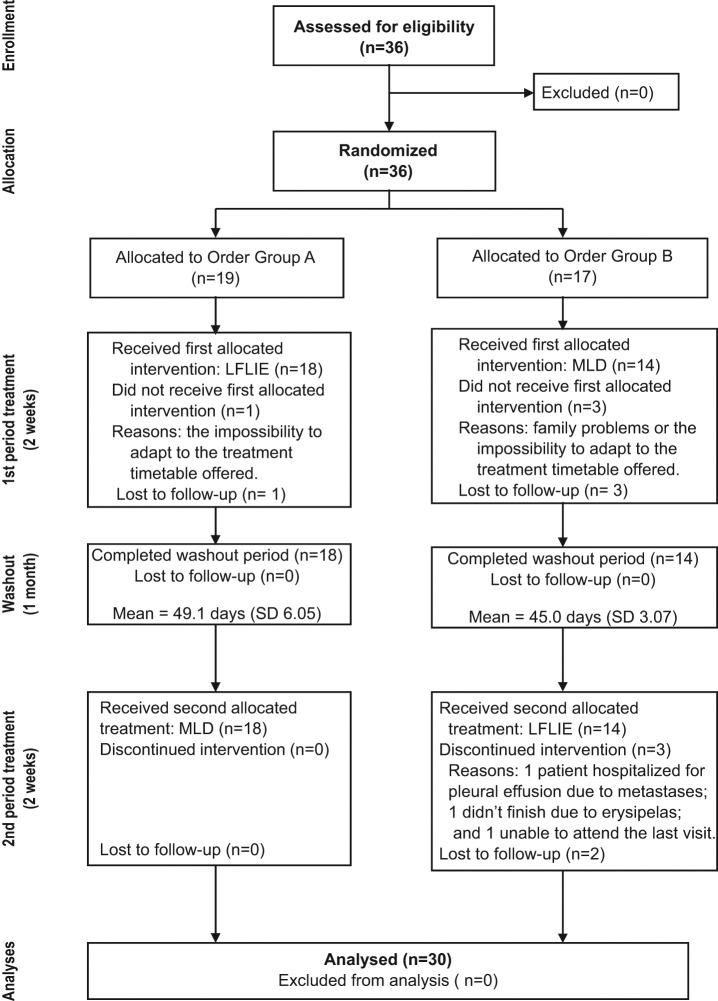
Flowchart diagram. MLD, manual lymphatic drainage; LFLIE, low-frequency low-intensity electrotherapy.

Figure 1 shows the flowchart. A total of 36 patients were randomized: 19 to group A and 17 to group B. A total of four patients declined to participate: one in group A and three in group B. Their reasons for refusing treatment were family problems or the impossibility to adapt to the treatment schedule. There were no cases of patients refusing to enter the study due to order allocation. All 18 group A patients who started the treatment completed it. In group B one patient only received nine sessions of the second treatment because she could not attend the 10th session for personal, not health-related, problems. This patient was evaluated after the ninth session and was included in the analysis. One patient from group B could not finish the second treatment because she had to be hospitalized for pleural effusion due to metastases. Another B group patient presented an episode of erysipelas in her upper limb lymphoedema during the second treatment but without the condition being examined. She informed us of this situation by phone but refused to come to the hospital to be evaluated. These two cases were lost to follow-up. A total of 12 group B patients finally completed treatment.

At recruitment, patients had a mean age of 67.8 years, and their average body mass index was 30.5 kg/m^2^. All patients had been surgically treated, 56% by breast-conserving surgery. All of them received axillary node dissection, while only four of them had previously been treated by sentinel lymph node biopsy. Almost all patients (87.5%) used regularly compression garments (four patients did not use garments because of skin problems or personal choice), and 46.9% had a history of infectious complications (Table 1).

**Table 1.** Demographic and medical characteristics of the sample with mean (SD) or *N* (%)

	Whole sample (*n* = 32)	Group A (*n* =18)	Group B (*n* =14)
Age (years)	67.78 (11.30)	69.56 (10.05)	65.50 (12.74)
BMI (kg/m^2^)	30.47 (6.31)	30.42 (6.65)	30.54 (6.09)
Surgical treatment			
Conserving surgery	18 (56.3%)	9 (50.0%)	9 (64.3%)
Mastectomy	14 (43.7%)	9 (50.0%)	5 (35.7%)
Sentinel lymph node biopsy (previously)	4 (12.5%)	2 (50.0%)	2 (50.0%)
Axillary node dissection	32 (100%)	18 (100%)	14 (100%)
Chemotherapy	25 (78.1%)	13 (72.2%)	12 (85.7%)
Radiation therapy	27 (84.4%)	15 (83.3%)	12 (85.7%)
Duration of lymphoedema (months)	73.22 (54.67)	87.56 (59.92)	54.79 (42.21)
Side of lymphoedema			
Right	15 (46.9%)	9 (50.0%)	6 (42.9%)
Left	17 (53.1%)	9 (50.0%)	8 (57.1%)
Lymphoedema severity			
Mild (<20%)	11 (34.4%)	6 (33.3%)	5 (35.7%)
Moderate (20–40%)	10 (31.3%)	6 (33.3%)	4 (28.6%)
Severe (>40%)	11 (34.4%)	6 (33.3%)	5 (35.7%)
Use of garments	28 (87.5%)	17 (94.4%)	11 (78.6%)
Infectious complications	15 (46.9%)	9 (50.0%)	6 (42.9%)

Table 2 shows the outcome results descriptive at each evaluation, as well as treatment effect for groups A and B separately. Treatment effect in the whole sample is showed in Table 3. When comparing the effects of low-frequency low-intensity electrotherapy and manual lymphatic drainage, there were no significant differences, except for a marginally significant reduction of pain which was greater with low-frequency low-intensity electrotherapy than with manual lymphatic drainage (13.1 vs. 1.07 mm) (*P* = 0.05).

**Table 2.** Mean (SD) of signs, symptoms and health-related quality of life scores at each evaluation by group of order

(a) Patients allocated to group order A						
	Baseline (*n* =18)	End of LFLIE treatment (*n* =18)	LFLIE Treatment effect (*n* =18)	Start of MLD Treatment (*n* =18)	End of MLD Treatment (*n* =18)	MLD Treatment effect (*n* =18)
Lymphoedema volume (mL)	845.6 (640.92)	841.5 (656.70)	−4.05 (89.98)	825.9 (623.97)	797.5 (643.72)	−28.36 (84.12)
VAS pain (mm)	16.22 (26.53)	8.44 (21.11)	−7.78 (16.87)	18.78 (29.56)	15.17 (28.19)	−3.61 (8.44)
VAS heaviness (mm)	37.17 (34.45)	22.39 (27.40)	−14.78 (20.92)	30.94 (31.38)	25.50 (30.89)	−5.44 (23.12)
VAS tightness (mm)	20.94 (27.22)	13.28 (24.57)	−7.67 (15.87)	22.17 (27.44)	16.28 (22.50)	−5.89 (15.38)
Health-related quality of life						
FACT-B+4 Subscales						
Physical well-being	21.50 (6.18)	22.55 (6.21)	1.05 (3.48)	21.55 (6.34)	22.28 (6.11)	0.73 (2.46)
Social well-being	18.97 (5.94)	19.45 (4.93)	0.47 (5.04)	17.30 (4.87)	18.94 (6.05)	1.64 (3.98)
Emotional well-being	18.06 (4.40)	18.38 (4.01)	0.31 (2.87)	16.77 (4.63)	17.21 (4.26)	0.44 (1.71)
Functional well-being	15.34 (5.68)	15.75 (5.03)	0.41 (3.46)	13.64 (5.58)	14.44 (5.32)	0.8 (2.4)
Breast cancer subscale	22.40 (4.48)	22.21 (5.65)	−0.19 (3.29)	22.01 (7.64)	22.04 (5.95)	0.03 (4.66)
Arm subscale	14.06 (4.74)	14.94 (5.24)	0.88 (2.26)	13.51 (4.93)	14.13 (4.64)	0.62 (1.96)
FACT-B+4 summaries						
FACT-General	74.33 (13.49)	77.15 (13.95)	2.83 (7.55)	69.83 (14.90)	73.48 (15.19)	3.65 (5.05)
FACT-Breast	96.65 (16.65)	99.57 (18.96)	2.92 (8.74)	91.65 (21.87)	96.08 (20.17)	4.43 (6.67)
FACT-TOI	59.85 (12.99)	61.94 (13.61)	2.1 (6.47)	57.89 (16.34)	60.16 (14.11)	2.27 (4.81)
MLD, manual lymphatic drainage; LFLIE, low-frequency low-intensity electrotherapy; VAS, visual analogue scale; TOI, trial outcome index.						
(b) Patients allocated to Group Order B						
	Baseline (*n* =14)	End of MLD treatment (*n* =14)	MLD treatment effect (*n* =14)	Start of LFLIE treatment (*n* =14)	End of LFLIE treatment (*n* =12)	LFLIE treatment effect (*n* =12)
Lymphoedema volume (mL)	718.7 (519.93)	677.4 (521.06)	−41.28 (98.99)	714.8 (651.55)	671.4 (552.47)	−43.34 (148.05)
VAS pain (mm)	19.75 (31.10)	22.50 (29.14)	2.75 (23.76)	34.92 (28.92)	13.83 (18.62)	−21.08 (34.2)
VAS heaviness (mm)	37.50 (34.08)	34.67 (34.43)	−2.83 (20.92)	35.75 (31.33)	17.33 (20.08)	−18.42 (29.79)
VAS tightness (mm)	33.75 (27.68)	33.00 (28.00)	−0.75 (27.6)	25.33 (29.37)	20.92 (22.40)	−4.42 (15.98)
Health-related quality of life						
FACT-B+4 subscales						
Physical well-being	22.67 (3.61)	22.00 (4.53)	−0.67 (3.61)	21.44 (5.27)	23.58 (4.12)	2.13 (3.5)
Social well-being	16.84 (7.34)	17.58 (8.65)	0.75 (3.1)	18.55 (7.60)	18.45 (7.38)	−0.1 (3.44)
Emotional well-being	13.89 (5.64)	14.78 (5.49)	0.89 (3.92)	12.33 (6.34)	16.56 (6.09)	4.22 (3.35)
Functional well-being	16.31 (3.66)	15.22 (6.72)	−1.09 (5.01)	13.85 (6.92)	17.67 (6.89)	3.81 (5.76)
Breast cancer subscale	20.94 (7.24)	18.23 (5.06)	−2.71 (3.86)	18.67 (6.12)	22.69 (8.23)	4.01 (5.37)
Arm subscale	12.00 (5.35)	12.60 (6.20)	0.6 (4.14)	12.10 (6.44)	15.30 (3.97)	3.2 (6.41)
FACT-B+4 summaries						
FACT-General	69.73 (11.86)	69.90 (14.14)	0.17 (9.95)	66.71 (14.76)	78.59 (17.45)	11.88 (10.55)
FACT-Breast	91.40 (18.39)	88.90 (17.02)	−2.5 (13.62)	85.83 (15.87)	102.96 (22.39)	17.13 (14.51)
FACT-TOI	60.28 (12.26)	55.88 (12.37)	−4.4 (9.86)	54.33 (11.99)	66.40 (13.87)	12.07 (12.93)

MLD, manual lymphatic drainage; LFLIE, low-frequency low-intensity electrotherapy; VAS, visual analogue scale; TOI, trial outcome index.

**Table 3.** Carry-over effect, period effect and treatment effect

	Carry-over effect		Period effect			Treatment effect				
	Mean [95% CI] of the difference between baseline and end of washout by group of order		Mean difference between treatments’ change by group of order			Mean change by treatment in the whole sample (*n* = 30)				
	Group A	Group B	Group A	Group B	*P*-value^1^	MLD	*P*-value^2^	LFLIE	*P*-value^2^	*P*- value^3^
Lymphoedema volume (mL)	−19.68 [−75.27, 35.92]	−3.94 [−103.8, 95.99]	−24.30	2.07	0.634	−33.52	0.048	−19.77	0.358	0.608
VAS pain (mm)	2.56 [−4.96, 10.07]	15.17 [−7.31, 37.64]	4.17	23.83	0.182	−1.07	0.723	−13.10	0.009	0.050
VAS heaviness (mm)	−6.22 [−14.31, 1.86]	−1.75 [−16.61, 13.11]	9.33	15.58	0.673	−4.40	0.281	−16.23	0.001	0.079
VAS tightness (mm)	1.22 [−9.36, 11.80]	−8.42 [−27.39, 10.56]	1.78	3.67	0.868	−3.83	0.322	−6.37	0.035	0.605
Health-related quality of life										
FACT-B+4 subscales										
Physical well-being	0.05 [−1.33, 1.43]	−1.22 [−3.83, 1.39]	−0.32	−2.80	0.245	0.26	0.643	1.41	0.044	0.188
Social well-being	−1.67 [−3.21, −0.13]	1.71 [−0.11, 3.53]	1.17	0.84	0.858	1.32	0.066	0.27	0.752	0.299
Emotional well-being	−1.29 [−2.94, 0.37]	−1.56 [−4.52, 1.40]	0.12	−3.33	0.140	0.60	0.268	1.72	0.023	0.265
Functional well-being	−1.70 [−3.69, 0.29]	−2.46 [−6.40, 1.48]	0.39	−4.91	0.109	0.12	0.868	1.64	0.089	0.238
Breast cancer subscale	−0.39 [−2.47, 1.69]	−2.26 [−5.78, 1.26]	0.22	−6.73	0.010	−0.95	0.276	1.31	0.138	0.085
Arm subscale	−0.54 [−1.79, 0.70]	0.10 [−2.84, 3.04]	−0.26	−2.60	0.459	0.61	0.281	1.74	0.046	0.334
FACT-B+4 summaries										
FACT-General	−4.50 [−7.62, –1.38]	−3.02 [−12.84, 6.81]	0.83	−11.71	0.046	2.49	0.096	5.84	0.006	0.197
FACT-Breast	−5.00 [−9.39, −0.60]	−5.56 [−18.65, 7.52]	1.50	−19.63	0.016	2.12	0.302	7.66	0.013	0.130
FACT-TOI	−1.96 [−5.67, 1.75]	−5.94 [−15.44, 3.55]	0.17	−16.47	0.028	0.04	0.977	5.42	0.015	0.074

MLD, manual lymphatic drainage; LFLIE, low-frequency low-intensity electrotherapy; VAS, visual analogue scale.

1. Unpaired *T*-test comparing the mean difference between treatments’ change of group A and B.

2. Paired *T*-test comparing pre- and post-treatment means.

3. Paired *T*-test comparing mean change of manual lymphatic drainage and low-frequency low-intensity treatment.

Pre-post low-frequency low-intensity electrotherapy treatment effect analysis (Table 3) showed a non-significant lymphoedema volume reduction of 19.77 mL (*P* = 0.36); pain diminished by a mean of 13.1 mm (*P* = 0.01); heaviness diminished by a mean of 16.23 mm (*P* = 0.001) and tightness diminished by a mean of 6.37 (*P* = 0.04). Also, the FACT-General, FACT-Breast and trial outcome index summaries increased significantly between pre and post low-frequency low-intensity electrotherapy treatment evaluations. None of the outcomes presented statistically significant changes between pre and post manual lymphatic drainage treatment evaluations.

In general there was no carry-over effect, except for some health-related quality of life scores in group A (Table 3). Similarly, a period effect was observed on some FACT scores, where group B presented significantly higher differences between treatment changes.

There were no reported adverse effects with manual lymphatic drainage treatment. As mentioned above, there was one patient with an episode of erysipelas on the fourth day of treatment with low-frequency low-intensity electrotherapy, who refused to come to the hospital for a control measurement until three weeks later, when the erysipelas had disappeared. On the third day of low-frequency low-intensity electrotherapy treatment, one patient presented erythema on the back of her hand which she attributed to a fold in her garment. When the garment was removed for two days the erythema disappeared and she continued with the treatment sessions. One patient showed skin irritation at an electrode point when receiving low-frequency low-intensity electrotherapy. When the intensity of the application was lowered, the problem was solved and she completed all the treatment sessions.

## Discussion

To our knowledge, this is the first clinical trial to compare the efficacy of low-frequency low-intensity electrotherapy with other breast cancer-related lymphoedema treatments. Although the benefits of low-frequency low-intensity electrotherapy on most symptoms and health-related quality of life were statistically significant, they were not significantly different from that of manual lymphatic drainage.

In terms of lymphoedema volume change, the effect of low-frequency low-intensity electrotherapy in our sample was negligible, as was the manual lymphatic drainage effect. Ricci^9^ reported a reduction of volume in 57% of their 28 upper limb secondary lymphoedema patients treated with low-frequency low-intensity electrotherapy, but there is no information about the amount of change. In a systematic review, Moseley et al.^4^ summarized the following lymphoedema volume reductions: 42% by manual lymphatic drainage and compression; 27% by complex decongestive therapy; 24% by manual lymphatic drainage; and 25% reduction by pneumatic pump therapy. Our findings differed substantially from these values because neither manual lymphatic drainage nor low-frequency low-intensity electrotherapy resulted in a volume reduction of more than 5%. However, most of these studies were focused on the initial reductive phase.

The fact that we did not obtain comparable lymphoedema volume reductions with low-frequency low-intensity electrotherapy nor with manual lymphatic drainage suggests that our sample could be different from those reported, probably because treatment was applied in the maintenance phase to a sample of previously treated patients with well-controlled lymphoedema. On the one hand, this homogenized the sample in order to avoid recently established lymphoedemas which are more responsive to treatment.^6^ But on the other hand, this could have limited the degree of the possible benefit obtained because all patients were properly controlled by periodical treatment sessions (once or twice per year) with manual lymphatic drainage. Moreover, most patients (28, 87.5%) regularly used compression garments, a strong factor in stabilizing lymphoedema volume in the maintenance phase.^18^

Lymphoedema has a negative health-related quality of life impact in breast cancer patients,^19^–^25^ so measuring this impact is considered important when evaluating treatments for breast cancer-related lymphoedema. In this trial, differences between both treatments on health-related quality of life changes were not statistically significant. However, the magnitude of change after low-frequency low-intensity electrotherapy was larger than after manual lymphatic drainage: the FACT-General improved by 5.8 vs. 2.5 points on average; FACT-Breast by a mean of 7.7 vs. 2.1 points; and trial outcome index a mean of 5.4 vs. 0.04 points. Furthermore, all these changes after low-frequency low-intensity electrotherapy treatment just meet the minimally important differences reported by Eton et al.^15^ (5–6, 7–8 and 5–6 points, respectively). With manual lymphatic drainage, the changes are far from these values. This is a rather paradoxical result: in our opinion, it suggests that low-frequency low-intensity electrotherapy could be effective in improving health-related quality of life, but there was not enough power to demonstrate differences with manual lymphatic drainage for secondary outcomes.

Pain, heaviness and tightness were significantly reduced after low-frequency low-intensity electrotherapy treatment, while after manual lymphatic drainage there were no significant differences in our sample. Nonetheless, differences between low-frequency low-intensity electrotherapy and manual lymphatic drainage for these symptoms were not statistically significant either. In any case, low-frequency low-intensity electrotherapy was as effective as manual lymphatic drainage in the analysed outcomes. Ricci^9^ reported that patients treated by low-frequency low-intensity electrotherapy improved in their ‘feeling of gravity and hardening’. As to Jahr et al.^8^ a significant decrease in pain (from 4.0 to 2.1) was reported for breast lymphoedema patients treated with very low-frequency low-intensity therapy combined with manual drainage.

In our study, the washout period worked adequately and there was no carry-over effect for most of the outcomes studied. Only some health-related quality of life summaries showed that the effect of low-frequency low-intensity electrotherapy treatment persisted after the washout period, but this effect was slight and the lowest limit of the 95% confidence interval (95% CI) was close to zero (–1.38 and −0.6). This means that a one-month washout time was long enough for the measurement of symptoms and volume. Williams et al.,^17^ in a cross-over study comparing manual lymphatic drainage with simple self-applied lymphatic drainage, used a washout period of six weeks, similar to our mean of 47.5 days.

In regard to adverse effects, we cannot be sure but we think that the episode of erysipelas was an accidental coincidence, though more attention should be paid to this point to confirm it. The intolerance to the electrode was easily solved by lowering the intensity of application and we considered it a minor problem. We therefore conclude that the treatment was well tolerated and there were no severe adverse effects.

There are some limitations to be considered in this trial. First, the small sample size and the fact that patients were not blinded for the treatment they received are the greatest potential bias problems. Second, selecting chronic lymphoedema patients limits the conclusion to only this chronic phase. The effect of low-frequency low-intensity electrotherapy in early lymphoedema and in the intensive phase of lymphoedema treatment would require future studies. Third, the study was designed to determine the immediate effect after 10 sessions of treatment with no further follow-up. Fourth, there was a period effect for several health-related quality of life outcomes studied. Both treatments showed additional benefits (higher changes) when administered after a previous one, but this additional benefit was greater for low-frequency low-intensity electrotherapy. This could indicate that combined treatments may result in more therapeutic benefits. Finally, the evaluated low-frequency low-intensity electrotherapy was associated with the maintenance methods that patients had been doing, such as compression garments. Compression is accepted for most authors as a measure of lymphoedema volume maintenance. Since there was very little information available about the effectiveness of low-frequency low-intensity electrotherapy, we believe that we should not expose patients to a worsening of oedema if they left maintenance treatment. In this context, it seems reasonable to assume that if patients did not change their maintenance treatment during the study, changes could be attributed to the added intervention.

Lymphoedema is a condition about which many aspects are still unknown, and for which there is no definitive treatment. The tolerance and acceptance of this new treatment was good. It supports the interest of further studies with low-frequency low-intensity electrotherapy to confirm the existence of beneficial effects in terms of health-related quality of life, and to identify which patients may obtain benefit from it (acute lymphoedema, lower limb lymphoedema, etc.).

In conclusion, this is the first study comparing low-frequency low-intensity electrotherapy with manual lymphatic drainage for several relevant outcomes (volume, pain, heaviness, tightness and health-related quality of life) in patients with chronic breast cancer-related lymphoedema. Although differences between both treatments’ changes were not statistically significant, the observed trend towards a better health-related quality of life is remarkable in low-frequency low-intensity electrotherapy. More studies are needed in order to assess that there is a possible new and effective therapy for lymphoedema patients, and at which phases of treatment the benefit could be highest.

**Clinical messages**

- There was no significant difference in outcomes between low-frequency low-intensity electrotherapy and manual lymphatic drainage treatments in chronic breast cancer-related lymphoedema.
- Tolerance and acceptance of this new treatment was good, and there was no relevant adverse effect.
- There is a trend towards a better health-related quality of life with the new treatment.
